# Client retention in the continuum of maternal health services in Ethiopia

**DOI:** 10.1186/s12913-023-09602-5

**Published:** 2023-06-02

**Authors:** Frehiwot Birhanu, Kiddus Yitbarek, Mirkuzie woldie

**Affiliations:** 1 School of Public Health, College of Health Science, Mizan-Tepi University, P.O.B. 260, Mizan-Aman, Ethiopia; 2 Department of Health Policy and Management, Faculty of Public Health, Jimma University, Jimma, Ethiopia; 3 Fenot Project, School of Population and Public Health, University of British Columbia, Addis Ababa, Ethiopia

**Keywords:** Continuum of care, EDHS, Ethiopia, Maternity care

## Abstract

**Background:**

Even though the global maternal mortality has shown an impressive decline over the last three decades, the problem is still pressing in low-income countries. To bring this to an end, women in a continuum of maternity care should be retained. This study aimed to assess the status of Ethiopian women’s retention in the continuum of maternity care with their possible predictors.

**Methods:**

We used data from the 2019 Ethiopian Mini-Demographic and Health Survey. The outcome variable in this study was retention in the continuum of maternity care, which consists of at least four ANC contacts, delivery in a health facility, and postnatal check within 48 h of delivery. We analyzed the data using STATA version 14 and a binary logistic regression model was used. In the multiple logistic regression model, variables with a *p*-value ≤ 0.05 were considered as significantly associated with the outcome variable. A weighted analysis was also done.

**Results:**

Of the 3917 women included in this study, only 20.8% of women completed all of the recommended services. Besides, the use of maternal health services favors women living in the biggest city administrations, followed by women living in agrarian regions; however, those living in the pastoralist area were disadvantaged. Having four or more ANC was explained by the maternal secondary level of education [AOR: 2.54; 95% CI: 1.42, 4.54], wealth status [AOR: 2.59; 95% CI: 1.45, 4.62], early initiation of ANC [AOR: 3.29; 95% CI: 2.55, 4.24], and being in a union [AOR: 1.95; 95% CI: 1.16,3.29]. After having four ANC, factor-affecting delivery in a health facility was wealth status [AOR: 8.64; 95% CI: 4.07, 18.36]. The overall completion of care was associated with women’s higher level of education [AOR: 2.12; 95% CI: 1.08, 4.25], richest wealth status [AOR: 5.16; 95% CI: 2.65, 10.07], timeliness of the first ANC visit [AOR: 2.17; 95% CI: 1.66, 2.85], and third birth order [AOR: 0.58; 95% CI: 0.35, 0.97].

**Conclusions:**

Despite the efforts by the Ethiopian government and other stakeholders, the overall completion of care was quite low. There is also a clear inequality because of women's background characteristics and regional variation. Strategies aiming to empower women through improved educational experience and economic standing have to be implemented in collaboration with other relevant sectors.

## Introduction

Even though the global Maternal Mortality Ratio (MMR) and neonatal mortality over the last two and three decades have shown an annual 2.9% and a 2.5% decline respectively [1, 2], the problem is still pressing in Low and Middle-Income Countries (LMIC). MMR is disproportionately higher in low-income regions: for instance, from the total pregnancy and childbirth-related maternal mortality, 7 out of 10 were from Sub-Saharan Africa (SSA) countries [3, 4]. As a result, the MMR in low-income countries is about 479 per 100,000 live births, whereas it is 41 per 100,000 live births in high-income countries [5]. In Ethiopia, about 112,000 newborn babies and 14,000 mothers die each year due to preventable causes [6, 7].

To bring this to an end, in 2015, the World Health Organization (WHO) launched a strategy to End Preventable Maternal Mortality (EPMM) through the maternal health service continuum across the stages of pregnancy, delivery, and postpartum periods [8]. By then, to meet the WHO's global target of reducing MMR to less than 70 per 100,000 live births on one side, and to lower neonatal mortality to less than 12 per 1000 Live births by 2030 on the other hand [9]. In Ethiopia, as part of the strategies, the government identified maternal, newborn, and child health as a priority agenda aiming to reduce the MMR from 412 to 42 per 100,000 live births by the end of 2035. In addition, with a thorough investment in maternal health services, neonatal mortality was planned to be reduced to 21 per 1000 live births in 2024/25 [10–12].

The maternity continuum of care is integrated care; to provide essential health care packages during pregnancy, childbirth, and postnatal periods. It is a focal point of health systems in many countries to minimize preventable maternal loss and complications in most instances and to make pregnancy and childbirth a positive experience in other cases [13]. The provision of the three integrated care including; antenatal care (ANC), delivery in a health facility, and postnatal care (PNC), as a continuum has gained global attention as one of the strategies to improve maternal and neonatal health outcomes [14]. As the evidence shows, complete coverage of the maternity continuum of care could avert an estimated 71% maternal mortality ratio (MMR) [15], and 56% of neonatal death worldwide [16].

As evidence suggests failing to obtain any of the care along the continuum is associated with discontinuity between maternal and child health programs which results in unfavorable maternal and neonatal outcomes [14, 17, 18]. Although studies show the completion of maternity services in Ethiopia, the findings are either limited to some geographic area/region, [15, 19], or the results present inconsistent figures ranging from 14 to 47% or focus on some services [15, 16, 20, 21]. In this paper, we set out to examine the degree of retaining clients within the continuum of maternity care in Ethiopia using recent nationally representative data. We also report on predictors of retention along the continuum of care and at completion.

## Method and materials

### Study setting and period

This study was conducted in Ethiopia, Africa’s oldest independent state situated in East Africa. Administratively, it is organized into seven agrarians (agriculture as the way of living) regions, two pastoralists (livestock raising as the way of living), two regions with both agrarian and pastoralist areas, and two chartered cities [22]. The population of Ethiopia is around 120 million, where nearly 25 million are shared by women of reproductive age [23]. The health system is federally decentralized along the eleven regions and two city administrations. Access to health care is an exigent challenge in Ethiopia, where around 5215 healthcare facilities managed by the public sector are there for the total population [24]. In addition, the health professional (Medical Doctors, Midwives, and Nurses) to population ratio was below the minimum requirement for SSA, 1.81 per one thousand population, in the year 2019 [25]. The study was conducted from March 21 to June 28, 2019.

### Population and sampling

In the study; we used data from the Demographic and Health Survey (DHS) database [26]. The sampling frame was a complete list of 149,093 enumeration areas (EAs) created for the 2019 Ethiopia Population and Housing Census (PHC). An EA is a geographic area covering an average of 131 households. The stratification made urban and rural areas for each region and administrative city, yielding a sum of 21 sampling strata.

Samples of EAs were selected independently in each stratum in two stages. In the first stage, 305 EAs (93 in urban areas and 212 in rural areas) were selected with probability proportional to EA size and with independent selection in each sampling stratum. A household listing operation was carried out in all selected EAs from January through April 2019. The resulting lists of households served as a sampling frame for the selection of households in the second stage [27].

Some of the selected EAs for the 2019 EMDHS were large, with more than 300 households. To minimize the task of household listing, each large EA selected for the 2019 EMDHS was segmented. Only one segment was selected for the survey, with probability proportional to the segment size. Household listing is conducted only in the selected segment, that is, a 2019 EMDHS cluster is either an EA or a segment of an EA. In the second stage of selection, a fixed number of 30 households per cluster were selected with an equal probability of systematic selection from the newly created household listing [27]. Women of the reproductive age group, who gave birth within 5 years preceding the survey, were the source population for this study.

All women of reproductive age group, either permanent residents of the selected households or visitors, were eligible for interview. In households with more than one eligible woman, one woman per household was randomly selected.

### Data collection

The survey team used DHS Program's standard tools that were adapted to reflect the population and health issues relevant to Ethiopia. Maternal health service-related information was collected using the women's questionnaire among the five questionnaires in the DHS program surveys. The questionnaire includes items related to respondents' background characteristics, reproduction, contraception, pregnancy and postnatal care, child nutrition, childhood immunizations, and health facility information. The Survey was implemented by the Ethiopian Public Health Institute (EPHI), in partnership with the Central Statistical Agency (CSA) and the Ethiopian Ministry of Health (MOH). Seventeen trainees who had some experience on previous Ethiopian DHS surveys obtained training from February 11–20, 2019, and proceeded to data collection after field practice and a debriefing session. The data was collected electronically using tablet computers [28].

### Study variables and measurement

#### Outcome variable

**Fig. 1:**
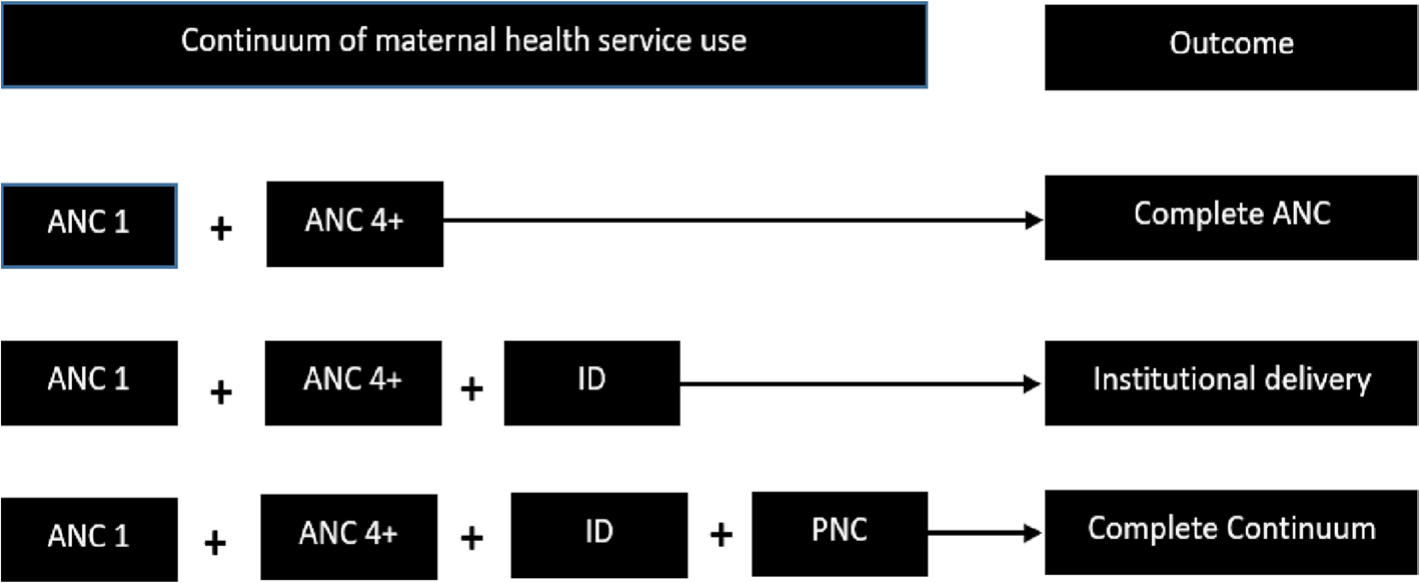
A structural presentation of maternal health service continuum of care for women aged 15–49 years, 2019 mini Ethiopian Demographic Health Survey, Ethiopia

The outcome variable in this study was the continuum of maternal health care. The continuum of care involves attending at least four ANC contacts, delivery in a health facility, and post-natal check within 48 h of delivery. The completion of maternal health service was declared if a woman obtained four or more ANC contacts, delivered in a health facility, and had a postnatal check within 48 h from delivery [29] [Fig. 1].

Four or more ANC means a woman who received four or above ANC contacts during her pregnancy according to the recommendation of the Ethiopian Ministry of Health during the study period [30]. The variable is a binary variable with two categories. We defined institutional delivery (ID) as a mother who gave birth in a health facility (private or public) with the necessary assistance. This variable was also a binary categorical variable. Similarly, PNC was also a binary variable categorized as 1, if the mother got check-ups within 48 h from delivery, and 0, otherwise.

#### Independent variables

To identify the predictors of the continuum of maternal health services in this analysis several independent variables were assesed. The independent variables include the age of the mother, the mother's level of education, birth order, place of residence (urban/rural), wealth quintile, the timing of the first ANC, mother's marital status, sex of the head of the household, history of child death, and mode of delivery.

#### Bias

To minimize the possible bias, missing response categories like “don’t know”, “Missing”, inconsistent” were excluded when calculating basic statistics during data analysis. In addition, all analyses were adjusted for cluster and sampling weights for disproportionate stratification of recruited participants.

#### Data analysis

We analyzed the data using STATA version 14. The analysis involved both descriptive and inferential statistics. Mostly, frequency and proportions were used to describe relevant characteristics of the study participants. We used a binary logistic regression model in two steps to identify predictors of completion in the continuum of maternity care. Primarily we did simple binary logistic regression for each of the independent variables. Variables with *p*-value ≤ 0.25 were candidates for inclusion in the multiple logistic regression models. Finally, a *p*-value ≤ 0.05 was used to declare the statistical significance of associations. We did a weighted analysis to account for disproportionate stratification for different sampling units, and to be able to generalize the findings to the national reference population.

## Results

### Description of the study participants

Around 3,979 women met the inclusion criteria, and 3,917 of them were included in the final analysis. Of the total women, the highest proportion (30.4%) were 25–29 years old, and most of them were married (93.8%). Coming to the place of residence, nearly three-fourths (73.9%) of them were rural dwellers. The highest proportion (51.3%), (21.0%) of them had no education at all and lies at the poorest wealth indices respectively. on the other side; most of them (86.6%), (77.9%) were male-headed households [HHs], and do not have a child's death history respectively [Table 1].

**Table 1:** The socio-demographic characteristics of the study participants, EDHS, 2019 (*n* = 3917)

**Variable**		**Continuum**		**Total**
	**Category**	**Complete f[%]**	**Not complete f[%]**	
Age	15–19	29[12.78]	198[87.22]	227[5.8]
	20–24	205[26.73]	562[73.27]	767[19.59]
	25–29	244[20.5]	946[79.5]	1190[30.4]
	30–34	163[20.48]	633[79.52]	796[20.33]
	35–39	110[18.68]	479[81.32]	589[15.04]
	40–44	43[16.73]	214[83.27]	257[6.56]
	45–49	19[21.35]	70[78.65]	89[2.27]
Marital status	Single	4[19.05]	16[76.19]	21[0.54]
	Married	769[20.92]	2907[79.08]	3676[93.85]
	Widowed	2[4.55]	41[93.18]	44[1.12]
	Divorced	38[21.59]	138[78.41]	176[4.49]
Sex of the child	Male	417[20.32]	1635[79.68]	2052[52.4]
	Female	397[21.3]	1468[78.76]	1864[47.6]
Residence	Urban	374[36.67]	646[63.33]	1020[26.04]
	Rural	440[15.19]	2457[84.81]	2897[73.96]
Mother's level of education	No education	242[12.04]	1768[87.96]	2010[51.31]
	Primary	322[22.82]	1089[77.18]	1411[36.02]
	Secondary	159[46.36]	184[53.64]	343[8.76]
	Higher	90[58.82]	62[40.52]	153[3.91]
Wealth quintile	Poorest	36[4.37]	787[95.63]	823[21.02]
	Poorer	107[13.02]	715[86.98]	822[20.99]
	Middle	112[14.72]	649[85.28]	761[19.43]
	Richer	182[25.89]	521[74.11]	703[17.95]
	Richest	377[46.72]	430[53.28]	807[20.61]
Sex of household head	Male	690[20.33]	2704[79.67]	3394[86.67]
	Female	124[23.75]	398[76.25]	522[13.33]
Ever had a child who died	No	696[22.79]	2358[77.21]	3054[77.97]
	Yes	118[13.67]	745[86.33]	863[22.03]

*F* Frequency

### The status of completion of maternal health services

**Fig. 2:**
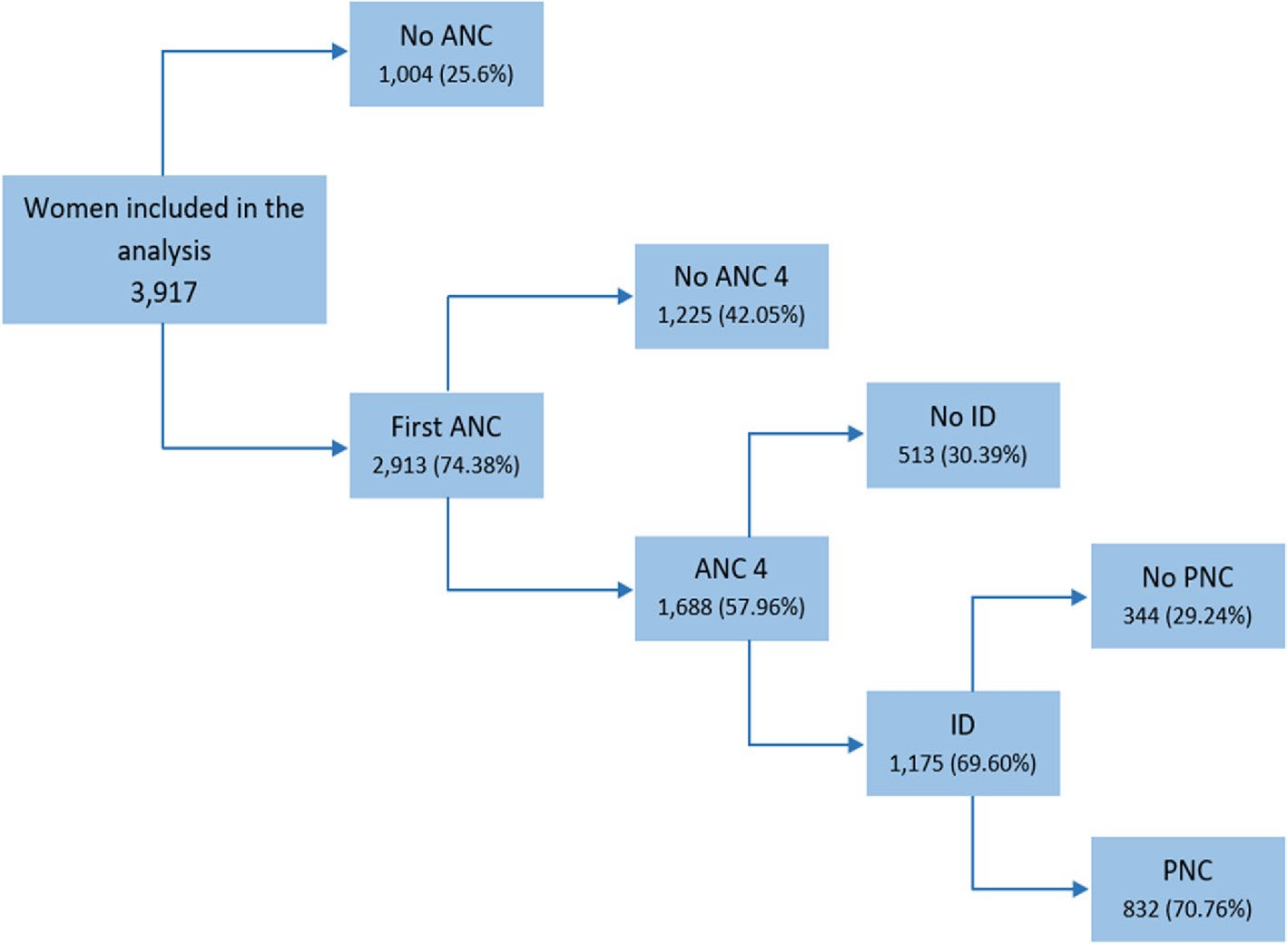
Decision tree depicting the degree of retention and dropout along the continuum of maternity care in Ethiopia, 2019 mini Ethiopian Demographic Health Survey

From the total 3,917 women included in the analysis, around three-fourths 2,913 (74.38%) of the women had their first ANC contact. From those who had the first ANC, more than half (57.6%) made it to the fourth or more ANC contacts; about 70% of women who had four or more ANC contacts gave birth in a health facility; furthermore, more than 70% of mothers who delivered in a health facility had PNC within 48 h of their delivery. Overall, 20.8% of pregnant women included in this study completed the continuum of care for their latest child [Fig. 2].

### The status of completion of maternal health services across regions

Completion of the maternal health service has shown to be higher in the capital city of Ethiopia, Addis Ababa, followed by the Tigray region and Dire Dawa city administration. The proportion of women who had first ANC contact is higher in Addis Ababa (96.8%), followed by Tigray and Gambella regions (94.7%), (86.3%) respectively. Sustaining the contact up to ANC 4 is similarly higher in Addis Ababa (85.4%), followed by Dirre-Dawa city administration, and Tigray regions. Women who had both at least 4 ANC contacts, and health facility delivery are likewise higher in Addis Ababa (97.7%), followed by Harrar, and Tigray regions. The status of completion for all services (ANC1, ANC 4, institutional delivery, and PNC) is higher in Addis Ababa (64.1%), followed by Tigray (45.2%), and Dire Dawa city administration (33.5%) [Table 2].

**Table 2:** The level of completion of maternal health services across the nine regions, and two city administrations, EDHS, 2019, Ethiopia

Region	> = 1 ANC (*n* = 3,917) **f[%]**	ANC 1 & 4 (*n* = 2,913) **f[%]**	ANC 1,4 & ID (*n* = 1,688) **f[%]**	ANC 1,4, ID & PNC (*n* = 1,175) **f[%]**	Continuum (*n* = 3,917) **f[%]**
Tigray	270[94.7]	183[67.8]	152[82.9]	129[84.9]	129[45.2]
Afar	32[62.8]	16[49.7]	10[61.6]	6[64.6]	6[12.4]
Amhara	711[84.9]	426[59.9]	298[69.9]	207[69.4]	207[24.7]
Oromia	1076[70.8]	617[57.4]	379[61.5]	233[61.4]	233[15.3]
Somali	63[29.2]	24[38.5]	14[58.7]	6[43.7]	6[2.9]
Benishangul	39[83.2]	26[67.5]	16[59.5]	11[73.2]	11[24.5]
SNNPR	558[71]	269[48.2]	185[69]	128[69.1]	128[16.3]
Gambella	16[86.3]	6[36.9]	5[83.2]	4[70.5]	4[18.7]
Harari	9[80.6]	4[48.6]	4[89.9]	3[71]	3[25]
Addis Ababa	121[96.8]	104[85.4]	101[97.7]	80[79.4]	80[64.1]
Dirre Dawa	18[84.3]	13[73.7]	11[85.5]	7[63.1]	7[33.5]
Total	2913[74.4]	1688[74.4]	1175[69.6]	814[69.3]	814[20.8]

#### Components of maternal health services

This study indicated that the use of maternal health services favors women living in the biggest city administrations followed by women living in mostly agrarian regions. Making 82.1% of the whole pregnant women, women living in city administrations had at least four ANC contacts. Early initiation of first ANC contact is correspondingly higher for women in the two city administrations (65.3%); followed by those living in pastoralist regions (46.1%). Almost all (94.8%) of women residing in the city delivered at the health facility, while this goes down by half (47%), and one-third (30.5%) for agrarian, and pastoralist women respectively. For around three-fourths (76.7%) of women living in the city post-natal check-up was made, whereas, similar was done for around one-third (35%), and only thirteen (25.6%) of agrarian, and pastoralist women respectively. Caesarian delivery service on the other hand has been applied for one-quarter of women living in the city administrations [Table 3].

**Table 3:** The level of completion of maternal health services among pastoralist, agrarian, and urban women, mini Ethiopian Demographic Health Survey, 2019, Ethiopia

Variable	Agrarian f[%]	Pastoralist f[%]	City f[%]	Overall (*n* = 3,917) f[%]
Four or more ANC	1569[41.9]	16[31.2]	104[82.7]	1688[43.1]
Initiation of ANC at 1^st^ trimester	995[36.4]	15[46.1]	79[65.3]	1088[37.7]
Delivered in a health facility	1757[47]	16[30.5]	119[94.8]	1891[48.3]
Assisted by the skilled birth attendant	1811[48.4]	18[35.4]	120[95.9]	1949[49.8]
Mode of delivery CS	212[5.7]	1[2.8]	31[24.5]	244[6.2]
PNC	1309[35]	13[25.6]	96[76.7]	1418[36.2]

*F* Frequency

The relation between the time of initiation of first ANC contact and the continuum of care.

**Fig. 3:**
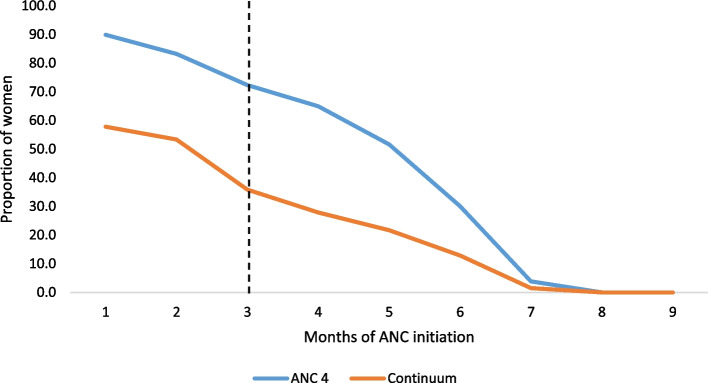
Time of ANC initiation versus completion of the continuum of maternity care among women aged 15–49 years, 2019 mini Ethiopian Demographic Health Survey, Ethiopia

As found in this study, for women who started their first ANC in the early 8 weeks, the chance of completion of the continuum was highest, and the chance of completion starts to go down as the weeks of first contact increased [Fig. 3].

### Factors associated with the status of the continuum of maternity care

Having four or more ANC was explained by mothers’ level of education. Women who attended secondary education were 2.5 folds higher (AOR: 2.54; 95% CI: 1.42–4.54) than uneducated women. Women's wealth status was the other factor for complete ANC attendance; richer women were 60% (AOR: 1.6; 95% CI: 1.11–2.32), and the richest women had two and a half folds (AOR: 2.59; 95% CI: 1.45–4.62) of a better chance of having complete ANC than the poorest women. Early initiation of the first ANC is the other factor having potential. As compared to women who were late to start their ANC, those who started earlier have three times (AOR: 3.29; 95% CI: 2.55–4.24) a better chance to attend four or more ANC. Finally, those who were in the union were two times (AOR: 1.95; 95% CI: 1.16–3.29) more likely to have four or more ANC attendance than women who were not in a union.

In our analysis, we found that after having four complete ANC the main factor affecting delivery in a health facility was wealth status. Institutional delivery among those who had at least four ANC was associated with the wealth status of the women. Especially the richest women were eight times more likely to deliver in a health facility (AOR: 8.64; 95% CI: 4.07–18.36) after having four complete ANC. The only factor we found affecting PNC after having four ANC and institutional delivery services is delivery by the caesarian section.

The overall completion of care was associated with women’s level of education. Those with secondary (AOR: 2.74; 95% CI: 1.24–6.07), and higher education, (AOR: 2.12; 95% CI: 1.10–4.25) were twice more likely to complete the course of care than uneducated women. The wealth status of the women was the other one. Compared to the poorest women, all other categories of wealth have a positive effect on completing a full continuum of care with an increasing pattern. For instance, the richest women had a five times more likelihood of completing all maternal health services compared to the poorest (AOR: 5.16; 95% CI: 2.65–10.07). Early initiation of the first ANC was found to have a positive effect on completing the maternal continuum of care (AOR: 2.17; 95% CI: 1.66–2.85). The birth order of the child is another factor affecting the continuum of care, whereby as compared to a woman with a child who was born first the chance of completion of care is about 42% less likely (AOR: 0.58; 95% CI: 0.35–0.97) for those who were born in the third order [Table 4].

**Table 4:** Predictors of completing the continuum of maternity care among women aged 15–49 years, EDHS, 2019, Ethiopia

Variables	Category	ANC > = 4	ANC > = 4 + ID	ANC > = 4 + ID + PNC	Continuum
		**(*n* = 3,962)**	**(*n* = 1,656)**	**(*n* = 1,202)**	**(*n* = 3,962)**
		**AOR[95%CI]**	**AOR[95%CI]**	**AOR[95%CI]**	**AOR[95%CI]**
Age group	15–19	1		1	1
	20–24	1.19[0.59,2.39]		2.07[0.69,6.19]	1.69[0.79,3.61]
	25–29	1.55[0.73,3.29]		1.02[0.37,2.81]	1.4[0.69,2.83]
	30–34	1.88[0.87,4.05]		1.54[0.54,4.35]	1.87[0.81,4.33]
	35–39	1.77[0.77,4.09]		1.28[0.42,3.92]	2.33[0.92,5.92]
	40–44	2.11[0.84,5.29]		2.23[0.54,9.29]	2.54[0.94,6.86]
	45–49	2.75[0.84,8.97]		3.73[0.32,42.76]	5.03[1.01,23.31]
Level of education	No education	1	1	1	1
	Primary	1.28[0.96,1.72]	1.05[0.68,1.64]	1.04[0.65,1.66]	1.25[0.9,1.75]
	Secondary	2.54[1.42,4.54]*	1.43[0.71,2.89]	1.39[0.65,2.97]	2.27[1.43,3.61]*
	Higher	1.85[0.92,3.7]	1.42[0.3,6.63]	1.94[0.8,4.7]	2.12[1.08,4.25]*
Birth order	1	1	1		1
	2	0.78[0.49,1.24]	0.75[0.39,1.43]		0.8[0.55,1.14]
	3	0.88[0.49,1.57]	0.32[0.18,0.6]		0.58[0.35,0.97]*
	4 or more	0.89[0.5,1.59]	0.4[0.23,0.71]		0.64[0.35,1.18]
Place of residence	Urban	1	1	1	1
	Rural	1.05[0.76,1.46]	0.85[0.44,1.64]	0.96[0.51,1.83]	0.96[0.59,1.54]
Wealth quintile	Poorest	1	1	1	1
	Poorer	1.6[1.04,2.44]	2.31[1.29,4.13]*	1[0.37,2.7]	2.22[1.31,3.75]*
	Middle	1.22[0.76,1.95]	2.34[1.18,4.65]*	1.23[0.39,3.88]	1.98[1.06,3.7]*
	Richer	1.6[1.11,2.32]*	4.57[2.25,9.26]*	1.59[0.56,4.52]	3.5[1.93,6.33]*
	Richest	2.59[1.45,4.62]*	8.64[4.07,18.36]*	1.53[0.53,4.4]	5.16[2.65,10.07]*
ANC 1 at first trimester	No	1	1	1	1
	Yes	3.29[2.55,4.24]*	1.03[0.71,1.47]	1.2[0.76,1.89]	2.17[1.66,2.85]*
Marital status	Currently not in union	1		1	
	Currently in union	1.95[1.16,3.29]*		0.66[0.2,2.18]	
Head of the household	Male		1		
	Female		1.36[0.72,2.57]		
History of child death	No	1	1		1
	Yes	1.08[0.76,1.52]	1[0.63,1.59]		1.07[0.66,1.72]
Birth by CS	No			1	1
	Yes			2.74[1.24,6.07]*	2.48[1.41,4.36]*
Constant		0.17[0.08,0.35]	1.25[0.54,2.89]	1.35[0.2,9.11]	0.06[0.02,0.12]

*denotes a *p*‐value of < 0.05

## Discussion

Reliant on the plausible benefit of completion of the recommended service during and after pregnancy, the maternity continuum of care got attention all over the world as a critical health intervention intending to improve both maternal and child health outcomes [31]. Respectively, the practice obtained recognition in Ethiopia a couple of decades back, and as a result maternal, and child mortality has reduced by half from the devastating Figure [32]. Despite the dramatic achievements from the lowest base, maternal death and morbidities are still stagnant which is partly attributable to the dropout of women from the recommended services during pregnancy, delivery, and post-partum periods. This study assessed women's level of accomplishment in care and the possible predictors of success.

The study found that of all women who gave birth within five years preceding the survey, only around one-fifth of them went through all the services during pregnancy, childbirth, and postpartum periods. This finding is in line with a report from the primary health care project in northern Ethiopia (21.6%) [21] and with a national study (21.5%) in the year 2019 [29]. It is revealed as there are improvements regarding completion of service as compared to previous studies like EDHS in 2016 [33], other local studies [34, 35], and SSA and South Asia [4, 36]. The improvement may be attributed to the Ethiopian government's and collaborators’ massive effort for implementing initiatives throughout the nation [37, 38]. Despite the progress made the figure is quite low compared to the findings in other developing countries like Pakistan (27%) [39], Zambia (38%) [18], Ghana (66%) [40], Cambodia (50%) implicating the need for an immense effort.

On the other hand, completion of the care had greater disparities across the regions, it was higher in the 2 city administrations; Addis Ababa (64.1%), Dire Dawa (33.5%), and Tigray region (45.2%), but it was unacceptably low in Somalia region (2.9%). This regional variation is similar to the synthesized information from previous studies in Ethiopia and other developing countries [33, 41–43]. The higher percentage of women who completed the continuum of care in the city is possibly due to the relatively better access to care so there is no way to discontinue the care due to transportation costs or, long walking hours to reach the facility [44]. In addition, formally employed women are abundant in urban areas, and most likely to be educated, which gives a woman better insight and understanding of the worthiness to follow the course. On the other hand, another national study indicated that despite the tremendous progress in health, it remains uneven between regions and cities [45].

Related to the above scenario attending the care at each spot has been shown to favor urban women more than those agrarian and pastoralists; obtaining the three services was like a fantasy for the pastoralist women. This finding is supported by earlier studies in Ethiopia [15, 46, 47]. In the pastoralist community, where cattle breeding is the main economic activity to support living, most people travel a lot to find grazing land and water for their cattle making accessing the health centers more challenging and [48] creating a bottleneck to care-seeking, and facilities available were poorly equipped. A study from one of the pastoralist areas in Ethiopia concluded that more than 85% of MM in the region were due to direct obstetric causes while according to the WHO estimate among all MMs around the globe, nearly 70% were attributable to direct obstetric causes [49]. This implies a higher burden of MM associated with the childbearing process in the pastoralist regions.

The overall completion of care was associated with women’s level of education. Those with secondary, and higher education were two times more likely to complete the course of care than those uneducated. This finding is commensurate with various earlier reports [50–53]. Education often plays a preventive role which gives women a positive influence to adhere to care either through enhancing their socio-economic status [54] or through boosting their knowledge and confidence.

Wealth status was a major characteristic, which had a strong effect on completing the continuum of care. Compared to the poorest, those in the other categories of wealth were more likely to go through all services, and the probability of service use increases as the wealth of the woman increases. Furthermore, wealth status is the only variable that had an effect at each step along the way to the continuum of maternity care. It affects ANC use, it also affects institutional delivery among those who had four or more ANC, and has an effect on the full continuum of maternity care. In studies conducted in Ethiopia, the pro-rich inequalities in maternal health are higher and are, even increasing compared to the previous period [55]. In addition, it is illustrated that in developing countries wealth-related inequalities in utilizing maternal health are overwhelming, especially in Asia and Africa, and yet are higher than inequalities for child vaccination [56, 57].

Another important issue in the completion of maternal health services is the timing of the initiation of the first ANC. Women who initiated the first ANC in the first trimester of pregnancy had more chance of attending all four recommended ANC contacts and completing the continuum of care. It is certain that if a woman starts the first ANC during the early pregnancy stages, she can get sufficient time to attend all the other ANC contacts so that she would have a better chance to complete the continuum. A similar study that was conducted in Kenya reported that women who initiated the ANC contact timely were three times more likely to complete all the recommended maternal health services [58].

On the other hand, the chance of completion of care in this study drops by half for the third child of the household compared to the first child. This finding is in line with what was found in the other three African countries [59]. This could be related to either a woman's encounter with inadequately person-centered care or a disrespectful experience during her prior health facility contacts, which may lead to an unfavorable perception about seeking care at a health facility during pregnancy and childbirth [60–63]. Alternatively, the women who had prior maternity care at a health facility may have the perception of having the required experience and knowledge to handle the circumstances surrounding pregnancy and childbirth [58]. 

## Limitations of the study

In this study, we used data from the DHS program database on the Ethiopian mini demographic and health survey with a limited number of variables. As a result, we could not recruit as many variables of interest as we intended.

## Conclusion and recommendations

Despite the efforts by all stakeholders, the overall completion of the maternity continuum of care is very low in Ethiopia. There was also region-to-region variation, where a higher completion of care in the two city administrations, Addis Ababa, Dire Dawa, and Tigray region. We have also found inequality of service use because of women’s background characteristics like level of education and wealth status. The significant dropout of those pregnant women who received the first ANC contact should alert maternal health program managers to find innovative approaches to retain women in the continuum. Such an approach shall aim to empower women through improved educational experience and economic standing by working with other relevant sectors. Furthermore, awareness creation interventions for the pregnant woman and her family would have the potential to raise early initiation of ANC. Lastly, future research has to investigate the reason behind higher regional variations in the continuum of care .

## Data Availability

The datasets generated and/or analyzed during the current study are available on the DHS program website. [https://www.dhsprogram.com/methodology/survey/survey-display-551.cfm].

## Abbreviations

ANC: Antenatal care
EA: Enumeration Area
EDHS: Ethiopian Demographic and Health Survey,
WHO: World Health Organization
LMIC: Low and Middle-Income Countries
MM: Maternal Mortality
MMR: Maternal Mortality ratio
PNC: Post-natal care
